# Draft Genome Sequence of *Halostagnicola* sp. A56, an Extremely Halophilic Archaeon Isolated from the Andaman Islands

**DOI:** 10.1128/genomeA.01332-15

**Published:** 2015-11-12

**Authors:** Sagar P. Kanekar, Neha Saxena, Soham D. Pore, Preeti Arora, P. P. Kanekar, P. K. Dhakephalkar

**Affiliations:** Bioenergy Group, MACS-Agharkar Research Institute, Pune, India

## Abstract

The first draft genome of *Halostagnicola* sp. A56, isolated from the Andaman Islands is reported here. The A56 genome comprises 3,178,490 bp in 26 contigs with a G+C content of 60.8%. The genome annotation revealed that A56 could have potential applications for the production of polyhydroxyalkanoate or bioplastics.

## GENOME ANNOUNCEMENT

A Gram-negative, pleomorphic, strictly aerobic bacterial strain was isolated from the sand of Elephant Beach, Havelock Island (part of the Andaman Islands) and designated A56. This extremely halophilic isolate showed optimum growth at 37°C and 20% (wt/vol) salinity and produced a significant quantity of polyhydroxyalkanoates (PHA), which can be used in the production of bioplastics. A56 exhibited the highest 16S rRNA gene sequence (1,452 nucleotides) homology with *Halostagnicola larsenii* XH-48 (99%). Whole-genome sequencing was performed to enhance a detailed understanding of the genome organization of this isolate and predict the presence of unique metabolic genes.

Genomic DNA was extracted using a GenElute Bacterial Genomic DNA isolation kit (Sigma, USA). The genome was sequenced using the whole-genome shotgun strategy using 316-chip and 200-bp chemistry on the Ion Torrent PGM platform (Life Technologies, USA). *De novo* assembly of the sequences was performed using MIRA assembler version 4.0.5 (1). Assembled data generated a genome containing 1 chromosome (4.13 Mb) and 4 plasmid sequences, which were filtered by homology-based alignment with the plasmid sequences (accession numbers- CP007056.1, CP007057.1, CP007058.1, CP007059.1) from the *Halostagnicola larsenii* XH-48 in MAUVE tool (2). Subsequently, 26 contigs representing 3.18-Mb genome sequence were obtained and annotated using NCBI’s Prokaryotic Genomes Automatic Annotation Pipeline (PGAAP) version 2.6. Gene prediction and functional characterization was performed with the help of the RAST server (3). The length of the largest contig was 712,144 bp (*N*_50_, 262,614). The annotation predicted 3,151 genes, including 2,718 coding sequences, 251 subsystems, and 45 total RNAs (4 rRNAs, 40 tRNAs and 1 other RNA gene). The RAST tool identified *Halogeometricum borinquense* DSM11551 as the closest phylogenetic affiliate of A56. Comparative genome analysis revealed a total of 131 unique genes associated with a subsystem (set of functional roles that make up a metabolic pathway, a complex, or a class of proteins) in A56. Furthermore, Digital DNA–DNA hybridization (4) revealed that A56 shared only 31.70% and 18.80% homology with *Halostagnicola larsenii* XH-48 and *Halogeometricum borinquense* DSM11551, respectively. These observations clearly differentiated A56 from its phylogenetic neighbors.

The A56 genome revealed the presence of genes coding enzymes such as polyhydroxyalkanoic acid synthase, 3-hydroxyacyl-CoA dehydrogenase, 3-ketoacyl-CoA thiolase, and acetyl-CoA acetyltransferase involved in biosynthesis of PHA, which can be used in the synthesis of bioplastics, the eco-friendly alternative of oil-derived plastics. A56 also harbored genes encoding biosynthesis of trehalose, as well as sodium/proton antiporters, which impart tolerance to halophiles against salt stress in hypersaline environment.

The whole-genome sequence has given insights into the metabolic adaptation of A56 to salt stress and has revealed the metabolic potential of A56 for the production of industrially important biopolymers.

### Nucleotide sequence accession numbers.

The draft genome sequence of *Halostagnicola* sp. A56 was deposited in the DDBJ/EMBL/GenBank database under the accession number JMIP00000000. *Halostagnicola* sp. A56 is available in the WDCM-recognized MACS Collection of Microorganisms, Agharkar Research Institute, Pune, India, under the accession number MCM B-1204.
